# Anastomosis for distal gastrectomy in Chinese patients: uncut roux-Y or roux-Y?

**DOI:** 10.1186/s12893-019-0672-8

**Published:** 2020-01-09

**Authors:** B. K. Sah, J. Li, C. Yan, C. Li, M. Yan, Z. G. Zhu

**Affiliations:** 1 Department of General Surgery, Gastrointestinal Surgery Unit, Ruijin Hospital Shanghai Jiao Tong University School of Medicine, 197 Ruijin Er Road, Shanghai, 200025 China; 2 Clinical Research Centre, Ruijin Hospital Shanghai Jiao Tong University School of Medicine, Shanghai, China

**Keywords:** Gastric cancer gastrojejunostomy, Uncut roux-Y, Roux-Y

## Abstract

**Background:**

An appropriate method of anastomosis is crucial for gastric cancer patients who require gastrojejunal anastomosis. Surgeons have proposed different types of modified gastrojejunostomies in the last two decades. We focused on two types of standard anastomosis, i.e., Uncut Roux-Y and Roux-Y gastrojejunostomies, and compared the differences in immediate postoperative complications between the two types.

**Methods:**

This is a retrospective study on 236 gastric cancer patients who underwent curative distal gastrectomy with gastrojejunal Roux-Y or Uncut Roux-Y anastomosis for six consecutive years. Immediate postoperative complications were compared between the two groups. The authors discussed the causes of the significant complications and their management.

**Results:**

There was no difference in demographics between the two groups (92 Roux-y Versus 144 Uncut Roux-y). The overall complication rate was 20.8% with 1.4% anastomotic leakage in the Uncut Roux-Y group versus 33.7% with 7.6% anastomotic failures in the Roux-Y group (*p* < 0.05). More abdominal infections occurred in the Roux-Y anastomosis group compared with the Uncut Roux-Y anastomosis group (*p* < 0.05). Duration of postoperative stay was significantly longer in patients with Roux-y anastomosis group (*p* < 0.05).

**Conclusions:**

Considering the surgical simplicity and postoperative complications, the Uncut Roux-Y is a better choice for anastomosis in patients with gastric cancer undergoing gastrojejunostomy. A well-designed large cohort in a multi-centre randomized controlled trial is necessary to support these findings and compare other aspects.

## Background

An appropriate anastomosis method is always critical in gastric cancer surgery, especially after distal gastrectomy. Surgeons prefer gastroduodenostomy (Billroth I) due to the simplicity of operative procedures and the similarity to the original anatomy and physiological conditions [1–3]. However, many clinicians also criticize this approach because of the increased reflux gastritis or oesophagitis [4]. There are also numerous studies on modified gastrojejunostomies, which are generally modifications of the typical Billroth II anastomosis [5].

Nevertheless, the essential components of these anastomoses were mainly grounded in jejunojejunostomy such that bile and pancreatic juices successfully passed through this bypass tract and the complications that arose from the remnant stomach were avoided. Moreover, surgeons mainly focused on diverting digestive juices to the jejunum to compensate for the so-called shortcomings of the Billroth II anastomosis. The most straightforward modification was the Braun jejunojejunostomy, which facilitates the diversion of digestive juices to the distal jejunum [5]. However, the reflux problem was still possible due to the continuity of both the efferent and afferent conduits of the jejunum to the remnant stomach. A milestone innovation was the Roux-Y gastrojejunostomy, which at the very least, anatomically solved the underlying problems of simple gastrojejunostomy plus jejunojejunostomy and was later also supported by Asian surgeons [6–8].

Nevertheless, the success of this modification was also challenged by Roux stasis syndrome and other functional complications, including stomal ulcers. A few studies also suggested that the neuromuscular discontinuity caused by jejunal dissection was the reason for these complications [9, 10]. Hence, on the grounds of this hypothesis, other modifications, such as the Uncut Roux-Y, surfaced as an effective alternative to the typical Roux-Y anastomosis [11, 12]. The main advantage of Uncut Roux-Y anastomosis was that it preserved the neuromuscular continuity of the jejunum and at the same time prevented the reflux gastritis or oesophagitis by tying the efferent loop of jejunum. And due to its simplicity, the Uncut Roux-Y was readily accepted by laparoscopic surgeons [13–15]. The main concerns regarding the selection of gastrojejunal anastomosis are summarized as three factors, i.e., the diversion of bile/pancreatic juices, the complexity of modified surgery and the postoperative complications. The postoperative complications are generally divided into two main types, i.e., immediate postoperative complications (in patient’s morbidity or any morbidity within one or 3 months after surgery) and delayed complications, which were generally focused on the quality of life of the patients. Few studies proposed the endoscopic evaluation system for post gastrectomy patients [16]. Due to the limitations of retrospective studies and availability of the data, we could only compare the immediate postoperative complications of the two types of standard anastomosis, e.g., Uncut Roux-Y and Roux-Y gastrojejunostomy.

## Methods

This study was a retrospective analysis, and the inclusion criterion was patients with gastric cancer who underwent curative distal gastrectomy with gastrojejunal Roux-Y or Uncut Roux-Y anastomosis. The primary endpoint was the presence of postoperative complications and any complications within 1 month of discharge from the hospital. The authors collected all data by comprehensively reviewing the original records of all the patients. Altogether, 236 patients were identified (144 Uncut Roux-Y and 92 Roux-Y). All patients underwent curative gastrectomies with appropriate lymph node dissections between January 2014 and July 2019.

There was no intentional bias for patient selection; all the patients for six consecutive years were included in the study. The authors collected detailed clinical parameters, including surgery type and the TNM classification of the tumour. All the complications were assessed and recorded blindly before the stratification and comparing the data between two groups. There were no significant differences in the clinical parameters between the two groups (Table 1). The authors recorded different types of complications in both groups (Table 2)

**Table 1:** Demographic

Parameter		Uncut Roux-Y	Roux-Y	*p* value
Age group (years)				0.208
≦50		20 (13.9)	15 (16.3)	
51–60		38 (26.4)	33 (35.9)	
61–70		63 (43.8)	28 (30.4)	
≧71		23 (16.0)	16 (17.4)	
BMI				0.559
< 20		25 (17.4)	11 (12.0)	
20–24		77 (53.5)	49 (53.5)	
25–29		36 (25.0)	29 (31.5)	
≧30		6 (4.2)	3 (3.3)	
Mode of surgery	Open	125 (86.8)	77 (83.7)	0.507
Laparoscopic		19 (13.2)	15 (16.3)	
TNM Stage				0.255
I		35 (24.3)	28 (30.4)	
II		27 (18.8)	10 (10.9)	
IIIA		28 (19.4)	13 (14.1)	
IIIB		27 (18.8)	17 (18.5)	
IIIC		27 (18.8)	24 (26.1)

**Table 2:** Postoperative complications between the two groups

Complication		Number of patients		*p* value
		Uncut Roux-Y	Roux-Y	
Overall complications		30 (20.8)	31 (33.7)	0.028
Abdominal complications		26 (18.1)	30 (32.6)	0.010
Haemorrhage	Intra-abdominal	0	1 (1.1)	0.390
	Anastomosis site	2 (1.4)	1 (1.1)	1.000
Wound dehiscence		1 (0.7)	2 (2.2)	0.562
Infection	Pulmonary	5 (3.5)	7 (7.6)	0.158
	Abdominal	7 (4.9)	12 (13.0)	0.024
	Central line	0	1 (1.1)	0.390
	Blood borne	0	3 (3.3)	0.058
	Wound	1 (0.7)	2 (2.2)	0.562
	UTI^a^	0	0	NA
Motility disorder (Need for NGT > 5 days)		6 (4.2)	2 (2.2)	0.488
Motility disorder		17 (11.8)	12 (13.0)	0.778
Anastomotic leakage		2 (1.4)	7 (7.6)	0.030
Impaired renal function		4 (2.8)	4 (4.3)	0.715
Respiratory failure		0	2 (2.2)	0.151
Cardiac		0	4 (4.3)	0.022
DVT^b^/PE^c^		0	0	NA
ICU admission		3 (2.1)	4 (4.3)	0.317
Readmission		8 (5.6)	4 (4.3)	0.770
Reoperation		2 (1.4)	3 (3.3)	0.381

^a^*UTI* Urinary tract infection, ^b^: *DVT* Deep vein thrombosis, ^c^*PE* Pulmonary embolism

### Surgical methods

**Fig. 1:**
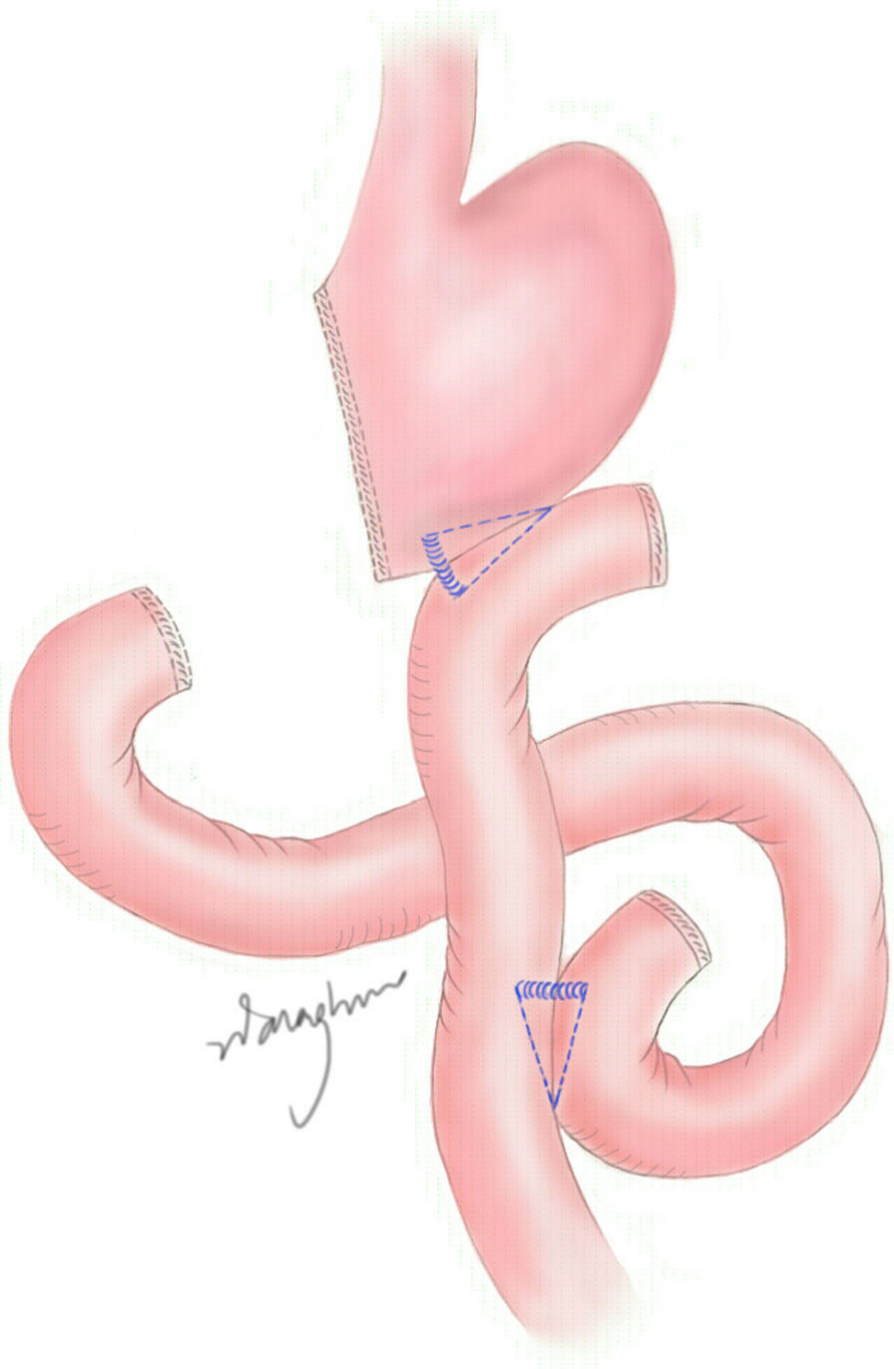
Typical Roux-Y gastrojejunostomy

**Fig. 2:**
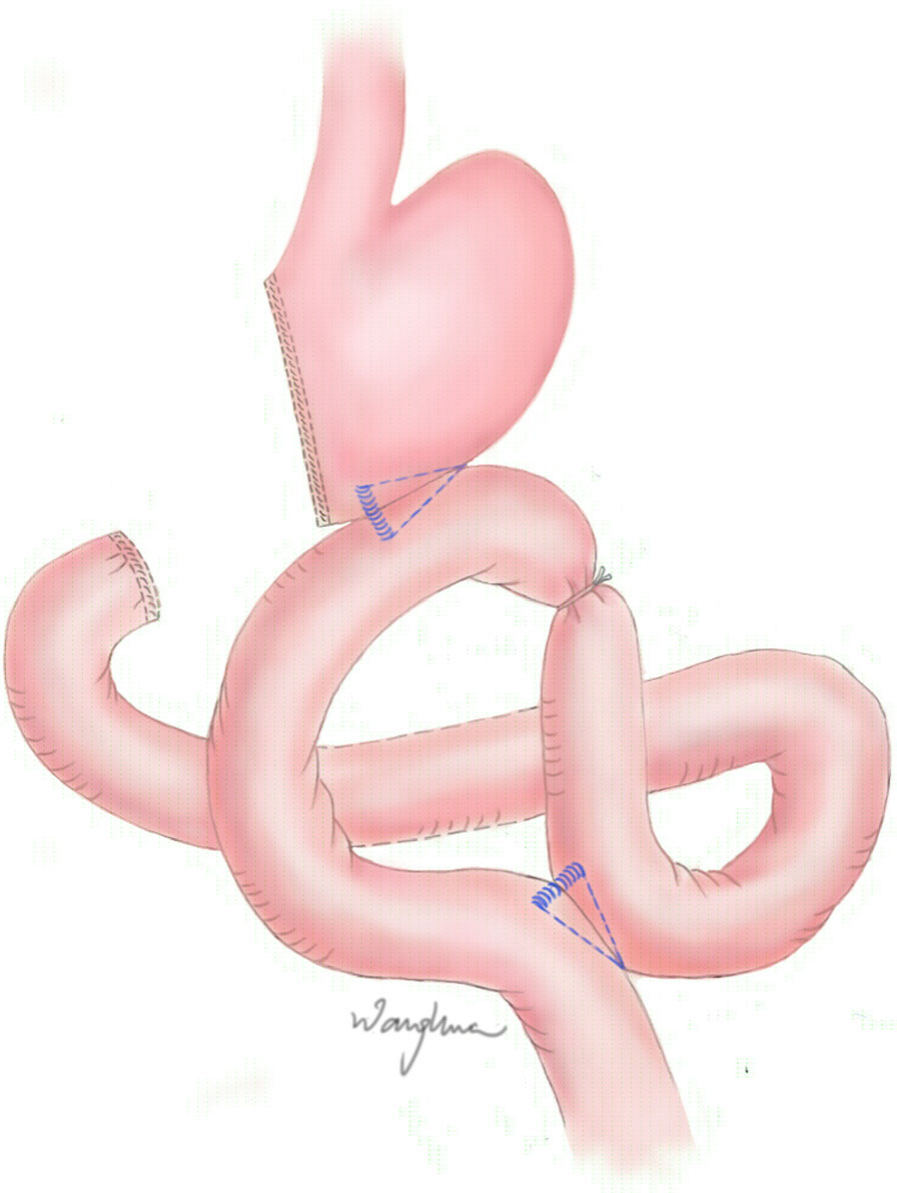
Uncut Roux-Y gastrojejunostomy

All patients underwent curative gastrectomy with appropriate lymph node dissection at the Department of Gastrointestinal Unit III in Ruijin Hospital, which is a specialized unit for gastric cancer and a well-known referral centre. Only the consultant surgeons with > 10 years of expertise in gastric cancer surgery were allowed to perform the operations mentioned above. Gastrojejunostomy with Roux-Y anastomosis was performed with linear staplers (Fig. 1). The duodenum was divided with a linear stapler and reinforced with external purse string sutures. The stomach was resected with a linear stapler at the appropriate margin from the tumour border. A continuous suture was applied for the reinforcement of the gastric wall. The jejunum was divided with a linear stapler at approximately 15 cm distal to the ligament of Treitz. The side to side anastomosis of gastrojejunostomy was performed with a linear stapler by approximating the greater curvature of the stomach and the distal jejunal loop. The joint hole of the gastrojejunal anastomosis was closed with continuous suture. Jejunojejunostomy was made with a linear stapler by approximating the proximal loop of the jejunum with the afferent loop of the jejunum at approximately 30 cm distal to the gastrojejunostomy. Similarly, the gastrojejunostomy with Uncut Roux-Y anastomosis was also performed with linear staplers; the only difference was that the jejunum was not divided and the efferent loop of the jejunum was tied approximately 3 cm distal to the gastrojejunostomy (Fig. 2). A thick silk suture (1–0 or 2–0) was passed through a silicon tube (inner diameter 1.o mm) to tie the efferent loop of jejunum. The Silicon tube was used to prevent the pressure injury on jejunum wall.

### Statistical analysis

The statistical analysis was performed with Statistical Package for Social Science (SPSS) version 22.0 for Windows (SPSS, Inc., Chicago, Illinois). Nonparametric methods were used to analyse data with an abnormal distribution. The chi-square test and Fisher’s exact test were used to compare the differences between the two groups as appropriate. A *p*-value of less than 0.05 was considered statistically significant.

## Results

### Types of complication

**Fig. 3:**
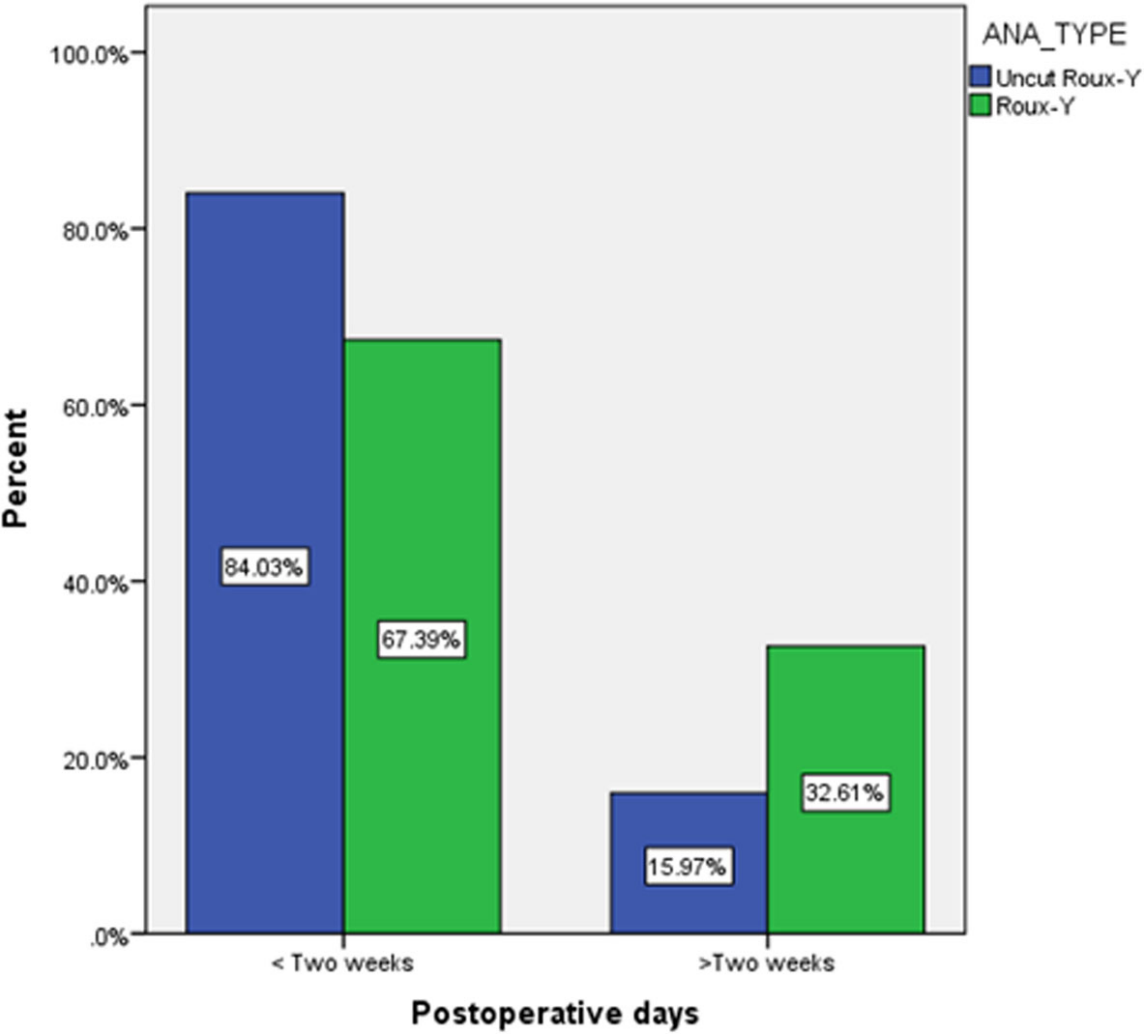
Difference of Postoperative stays at hospital

The authors compared the occurrence of complications between patients with Roux-Y and Uncut Roux-Y anastomoses (Table 2). There was significant difference in the overall postoperative complications, anastomotic leakage, abdominal infection and cardiac failure rates between the two types of anastomosis methods (*p* < 0.05). About 84% of patients in Uncut Roux-Y group were discharged in less than 2 weeks after a smooth recovery and removal of suture. Duration of postoperative stay was significantly longer in patients with Roux-y anastomosis group (Fig. 3, *p* = 0.003). About 33% of patients in Roux-Y anastomosis group stayed at hospital for more than 2 weeks after surgery which was almost double of that in Uncut Roux-Y group.

The clinicians observed more anastomotic leaks in patients with the Roux-Y anastomosis compared with patients with the Uncut Roux-Y anastomosis (seven versus two, *p* = 0.030). None of the patients died of postoperative complications.

### Site of anastomotic leakage and management

Rupture of the duodenal stump was the main cause of anastomotic failure. Among the nine patients with an anastomotic leak, only three patients underwent reoperation, and the remaining six patients were managed by conservative methods, mainly by continuous irrigation of the abdominal drainage tube (Table 3). Six patients with anastomotic leakage eventually had digestive content in the drainage tube, which suggests that an adequate draining system at the anastomosis site was crucial not only for diagnosis but also for treatment.

**Table 3:** Sites of anastomotic leakage and management strategies

Anastomosis	Site of anastomosis leak	No.	Method of diagnosis	Treatment
Uncut Roux- Y	Gastric lesser curvature	1	Reoperation	Reoperation
	Duodenal stump	1	Reoperation	Reoperation
Roux -Y	Duodenal stump	4	Digestive content in drainage tube	Continuous irrigation
	Duodenal stump	1	Digestive content in drainage tube	Reoperation
	Gastrojejunal anastomosis	1	Digestive content in drainage tube	Continuous irrigation
	Gastrojejunal anastomosis	1	Reoperation	Reoperation

## Discussion

There have been various studies on the operative techniques for gastric cancer surgery, especially laparoscopic gastrectomy, which has gained notable popularity in various centres in Asian countries over the last two decades [17–19]. Few randomized control trials have supported the generalization of laparoscopic surgery for gastric cancer to surgery for advanced-stage gastric cancer [20]. Even robotic surgery has received reasonable attention in this field [21]. Nevertheless, regardless of the mode of surgery, the fundamental concern of oncological surgeons was still the same, e.g., appropriate resection of the stomach and dissection of regional lymph nodes. One of the crucial factors that drew the continuous attention of surgeons was the method of anastomosis. The selection of the anastomosis was mainly focused on decreasing the postoperative complications and quality of life. Due to the limitations of retrospective study and availability of the data, we could only compare the immediate postoperative complications of the two types of standard anastomosis, namely, Uncut Roux-Y and Roux-Y gastrojejunostomy. The main cause for the missing data on the quality of life was the inconsistency of the patient’s follow-up. There was no centralized follow-up centre for these patients; patients generally visited their operating surgeons a month after surgery. And there was no recorded data of any follow up findings. This was a serious limitation of this study. A better prospective study design is necessary to address this problem.

Moreover, many patients did not return for future follow-up at the same hospital. Even at the same hospital, the follow-up plans varied among different surgeons. In addition, the gastroscopy was performed by endoscopists, and the reports were mainly focused on whether there was tumour recurrence. In the retrospective study of the last 3 years, the data on quality of life, gastroscopy evaluation of the anastomosis and reflux gastritis was not able to be collected.

In general, different scales of postoperative complications are anticipated for the different stages of primary tumours; more postoperative complications were observed in late-stage gastric cancer [22]. Therefore, the data were checked for proportion of patients with different stages, which increased the reliability of this retrospective study. To minimize the selection bias, the authors also compared the patients according to laparoscopic and open surgery (Table 1). The authors compared immediate postoperative complications between the two groups, and there were statistically significant differences in the complications rates between the two groups, especially in the infectious complications and anastomotic leak.

Authors did not find any definite cause behind higher incidence of anastomotic leak in Roux-Y group; it might be simply because of inadequate sample number in this study, as we know the anastomotic leak rate is very rare postoperative complication. Hypothetically, the division of jejunum in Roux-Y group would cause motility disorder or Roux stasis syndrome. Therefore, the increased pressure at duodenal stump would be the reason for rupture and it would have caused by poor motility of distal jejunum. However, authors also noticed that there was no significant difference of motility disorder between two groups. It might be that surgeons were already aware of roux stasis syndrome and they took proper preventive methods during surgery, such as size of remnant stomach (relatively small in size) and adequate length of distal limb of jejunum (30 cm from gastrojejunal anastomosis to jejuno-jejunal anastomosis). Besides, it is very hard to identify the motility disorder or even the roux stasis syndrome in retrospective study, simply because of inaccurate documentation in patients’ history book. Furthermore there is no standard definition for these functional complications and very difficult to diagnose it correctly. Therefore we tried to give an objective discrimination and separately listed patients who had motility disorder and warranted gastrointestinal decompression through nasogastric tube for more than 5 days. Five days was simply set empirically to give a general idea on severity of motility disorder.

Another advantage of the Uncut Roux-Y anastomosis was that the surgeons needed fewer linear staplers, thus decreasing the overall expenditure. Moreover, even if the postoperative complications of the Uncut Roux-Y group were similar to those of the Roux-Y group, Uncut Roux-Y anastomosis should still be the first choice due to its simplicity and cost-effectiveness. Nevertheless, these data represent the results of six consecutive years at a specialized centre for gastric cancer surgery in a Chinese hospital, and at the very least, and these data support the previous studies in other centres that demonstrated the Uncut Roux-Y is an excellent method of anastomosis for distal gastrectomy, which is feasible as a laparoscopic surgery [23, 24].

There are few limitations too, it is possible that the ligature was not effective and the closure failed at efferent loop of jejunum in Uncut Roux-Y patients. The best way to recognize the problem is through endoscopic or radiological confirmation after intake of oral contrast agent. But, as we mentioned this was a retrospective study and we do not have such data to elaborate it. However, it is also very logical that even if the closure was not hundred percent effective but this method would prevent the reflux of large amount of bile or pancreatic juice comparing to simple gastrojejunostomy. For symptomatic patients, anti reflux medicines are prescribed for such problems. Similarly, due to retrospective nature of this study, there would be concern of patient selection bias or how the complications were assessed. Thus the detection of the outcome could have differed in each group. The authors declared that there was no intentional bias for patient selection.

## Conclusion

Considering the surgical simplicity and postoperative complications, especially the fewer anastomotic leak and shorter postoperative stay at hospital compared with Roux-Y anastomosis. The Uncut Roux-Y is a better choice for anastomosis in patients with gastric cancer undergoing gastrojejunostomy. A well-designed large cohort in a multi-centre randomized controlled trial is necessary to support these findings and compare other aspects, especially the reflux rate and quality of life after surgery.

## Data Availability

The datasets used and/or analysed during the current study are available from the corresponding author upon reasonable request.
